# Influence of Light-Intensity-Dependent Droplet Directionality on Dimensions of Structures Constructed Using an In Situ Light-Guided 3D Printing Method

**DOI:** 10.3390/polym14183839

**Published:** 2022-09-14

**Authors:** Jongkyeong Lim, Sangmin Lee

**Affiliations:** 1 Department of Mechanical Engineering, Gachon University, Seongnam 13120, Korea; 2 Division of Mechanical, Automotive and Robot Component Engineering, Dong-Eui University, Busan 47340, Korea

**Keywords:** droplet directionality, high-speed imaging, image analysis, layer thickness, light guide, light intensity, structure diameter, support-free 3D printing

## Abstract

As an alternative to conventional 3D printing methods that require supports, a new 3D printing strategy that utilizes guided light in situ has been developed for fabricating freestanding overhanging structures without supports. Light intensity has been found to be a crucial factor in modifying the dimensions of structures printed using this method; however, the underlying mechanism has not been clearly identified. Therefore, the light-intensity-dependent changes in the structure dimensions were analyzed in this study to elucidate the associated mechanism. Essentially, the entire process of deposition was monitored by assessing the behavior of photocurable droplets prior to their collision with the structure using imaging analysis tools such as a high-speed camera and MATLAB^®^. With increasing light intensity, the instability of the ejected falling droplets increased, and the droplet directionality deteriorated. This increased the dispersion of the droplet midpoints, which caused the average midpoints of the deposited single layers to shift further away from the center of the structure. Consequently, the diameter of the structure formed by successive stacking of single layers increased, and the layer thickness per droplet decreased. These led to light-intensity-dependent differences in the diameter and height of structures that were created from the same number of droplets.

## 1. Introduction

The use of a support layer—commonly known as a support—in typical 3D printing methods, such as polyjet technology [1,2,3], stereolithography [4,5,6], fused deposition modeling [7,8,9,10,11], and selective laser sintering [12,13,14], is imperative to the accurate and stable fabrication of products with an overhanging geometry [15,16,17,18]. However, the use of the support causes various problems such as the need for additional materials, an increase in manufacturing time due to process complexity, and an increase in the probability of error [18]. Additionally, the removal of the support via physical/chemical routes after the fabrication of the final product causes side effects such as damage or contamination of the product surface and structural deformation [17,18,19]. To overcome these limitations of conventional 3D printing methods, a new 3D printing strategy utilizing in situ guided light has been conceptualized and demonstrated by our research group for accurate and stable fabrication of freestanding overhanging structures without supports [20]. The new 3D printing strategy is inspired by the in situ light-guiding characteristic of optical fibers that is based on total internal reflection at the interface between the core and cladding [21,22,23]. The hydrogel-based printed structure and air function as the core and cladding, respectively (refractive indices of ≈1.47 [23] and ≈1.00 [24], respectively). The light irradiated from the laser is guided and transmitted into the hydrogel-based structure by total internal reflection, thereby the hydrogel-based structure itself becomes a light source. Droplets continuously ejected through an on-demand pneumatic printing system harden as soon as they collide with the structure, forming single layers. Therefore, freestanding overhanging structures without supports can be formed by successive layer-by-layer stacking. This new approach has thus far been used to fabricate mainly cylindrical structures; its applicability to fabricate other types of structures is being investigated. In the case of cylindrical structures, a seed structure, which is the basis of a main structure, is initially formed. The seed structure grows while gradually decreasing in diameter, and after reaching a specific diameter, the main structure having the same diameter is continuously formed. The dimensions and shape of main structures fabricated using this method depend on various factors such as the light intensity, characteristics of photocurable droplets (such as viscosity, surface tension, and degree of polymerization), conditions of the dispensing system (such as nozzle size and pressure), as well as environmental parameters (such as temperature and humidity). In particular, the light-intensity-dependent changes in the behavior of the photocurable droplets are critical in determining the possible dimensions of the main structure. In a previous study regarding identification of geometric range of main structures that can be fabricated using the light-guided 3D printing method [25], our research group analyzed the effects of light intensity on the diameter of the main structure and the layer thickness per droplet. The diameter of the main structures increased from 0.9 to 1.2 mm and the layer thickness per droplet decreased from 100 to 60 μm with increasing light intensity from 50 to 300 mW cm^−2^. These parameters were found to be significantly affected by the fluidity of the photocurable droplets, which decreased with increasing light intensity, and the surface area on the top of the main structure. In particular, the surface area on the top of the main structure has a greater effect on the dimensions of structures than the fluidity of the droplet. As the diameter of the main structure increases, the surface area through which the droplet can spread increases to ensure that it extends until it covers the surface. Because the layer thickness per droplet is inversely proportional to the spreading area, the layer thickness realized per droplet decreases with increasing light intensity. This leads to a difference in the height of structures that are fabricated from the same number of droplets. Despite these analyzes, the previous study was restricted owing to its focus only on analyzing the deposition after the droplets collided with a main structure without analyzing the reason why the main structure had the corresponding diameter with light intensity.

In this study, the light-intensity-dependent changes in the main structure dimensions were analyzed by (1) measuring the variation in the directionality of the droplets before they collided with the main structure as well as the seed structure, and (2) monitoring the formation of the seed and main structures after the droplet collision. To this end, the effects of light intensity on the directionality of the continuously ejected droplets were investigated by analyzing the midpoints of the droplets immediately before their collision with the seed and main structures. Subsequently, the effects of the droplet directionality on the shape and position of the deposited single layers were investigated by analyzing their midpoints. Through these analyzes, the effect of light intensity on the dimensions of main structures is clearly identified, and ultimately, the dimensions of main structures that can be fabricated and manipulated with the light-guided 3D printing method can be defined.

## 2. Materials and Methods

### 2.1. Materials

A photocurable hydrogel precursor solution was prepared by mixing 90% *v v*^−1^ poly (ethylene glycol) diacrylate (PEGDA, 575 Da) in 10% *v v*^−1^ deionized water. Subsequently, a water-soluble visible-light photoinitiator (Eosin Y; 0.1 × 10^−3^ M), co-initiator (triethanolamine; TEA; 1.5% *w v*^−1^), and catalyst (1-vinyl-2-pyrrolidinone; NVP; 1.0% *w v*^−1^) were added to the preceding solution. All materials used to prepare the hydrogel precursor solution were purchased from Sigma-Aldrich. In terms of rheological characteristics, the prepared photocurable hydrogel precursor solution had a kinematic viscosity of 40–60 mPa (corresponding to shear rates of 0–1000 s^−1^) at 25 °C and a surface tension of approximately 38 mN m^−1^ [25].

### 2.2. Experimental Setup

The light-guided 3D printing system consists of a pneumatic printing system [26], a 532 nm laser (CNI, MGL-III-532, Changchun, China), silver mirrors (Thorlabs, PF10-03-P01, Newton, NJ, USA), a Z-axis motorized stage (Sciencetown, SM4-0830-4S, Incheon, Korea), and a glass substrate (SciLab, SL.Cov1037, Versailles, France). The pneumatic printing system enables continuous dispensing of a high-viscosity hydrogel precursor solution as single droplets on-demand. The orientation of the silver mirrors can be adjusted to permit irradiation of the light emitted from the 532 nm laser onto the glass substrate at a desired position. The ejection of individual droplets (2 Hz) through the pneumatic printing system and the on–off sequence of the laser were programmed using LabVIEW software (National Instruments, Austin, TX, USA). The volume and velocity of each ejected droplet were fixed at ≈70 nL and ≈1.1 m s^−1^, respectively. Additionally, the distance between the top of the hydrogel-based structure and the tip of the printing head (i.e., the standoff distance), which is a crucial factor for enabling clogging-free droplet discharge from the nozzle, was maintained at 2 cm during the entire printing process.

### 2.3. Image Analysis

The behavior of each droplet and the structure before/after the droplet collision were examined using a high-speed camera at 30,000 frames per second (Fastcam Mini AX100, Photron, Tokyo, Japan). Based on the images acquired using the high-speed camera, the shapes and midpoints of the droplets and the formed single layers as well as the combined images of the formed single layers were corrected and analyzed using an image processing toolbox in MATLAB^®^ (MathWorks, Natick, MA, USA).

## 3. Results and Discussion

### 3.1. Light-Intensity-Dependent Changes in Dimensions of a 3D-Printed Structure Using an In Situ Light-Guided Method

The operation of the light-guided 3D printing method is described henceforth. The method can be used to fabricate any cylindrical specimen, but a linear structure was considered for the purpose of the study. First, light emitted from a laser is reflected through mirrors and focused onto a glass substrate at a desired position. Then, the photocurable hydrogel is ejected by a pneumatic printing system onto the desired location of the glass substrate in the form of droplets, which are directly cured by light to form a hydrogel-based structure. The light received from the laser is guided by the hydrogel-based structure, which acts as a light source for the next ejected droplet. Then, the next droplet is instantly polymerized by the guided transmitted light immediately after it collides with the structure. Consequently, the final designed hydrogel structure can be fabricated by repeatedly performing the ejection–collision–curing process without a support. Additional details regarding the operation of the light-guided 3D printing method can be found elsewhere [20,25]. In the light-guided 3D printing method, light intensity is an important factor in determining the dimensions of the printed structure, such as the diameter of the main structure or the thickness of the layer deposited per droplet. 

**Figure 1:**
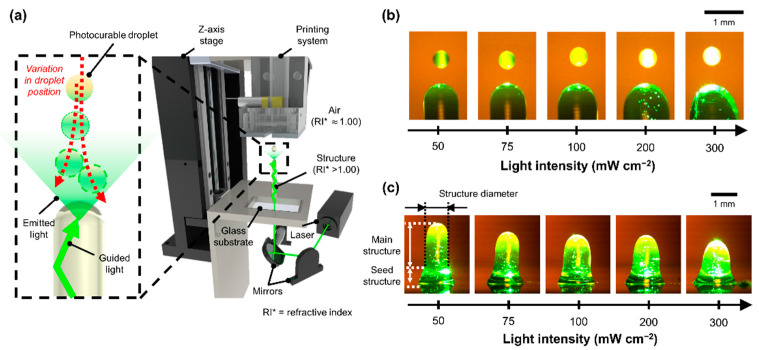
Light-intensity-dependent changes in dimensions of the structure. (**a**) Schematics of the investigated 3D printing system and the variation in droplet position; (**b**) effects of different light intensities irradiated onto the droplets, and (**c**) the light-intensity-dependent changes in structure dimensions.

The variation in the final collision point of each ejected droplet was presumed to increase with increasing light intensity (Figure 1a); this phenomenon was considered to be primarily responsible for the light-intensity-dependent changes in droplet directionality. The directionality of each ejected falling droplet, which is a measure of its linear movement, presumably deteriorates with increasing amount of light energy exposed to it. This change in directionality can be ascribed to the increased instability in the movement of the hydrogel droplets due to their non-uniform curing during motion. In general, each ejected falling droplet undergoes an oscillatory motion, that is, contraction and expansion [27,28,29,30]. Each falling hydrogel droplet is cured by the irradiated light; however, non-uniform curing may occur inside the droplet owing to a misalignment of the optical path between droplets and the rate of light energy absorbed by each droplet. Fluid properties such as density and viscosity vary non-uniformly at each location in the droplet owing to the short-duration curing, and the non-uniformity of the droplets is exacerbated with increasing light intensity (Figure 1b). As the extent of non-uniformity of the droplet increases, the instability of the droplet motion increases, which changes the directionality of the droplet, thereby leading to a greater variation in the deposition positions with respect to the final surface. With increasing light intensity more droplets were deposited on the point further away from the target point, which was presumably the factor responsible for the increase in the diameter of the structures during the formation of the structure (Figure 1c). To prove this hypothesis, the position of the photocurable droplets immediately before they collided with the structure was analyzed according to the light intensity.

### 3.2. Light-Intensity-Dependent Directionality of Droplets

**Figure 2:**
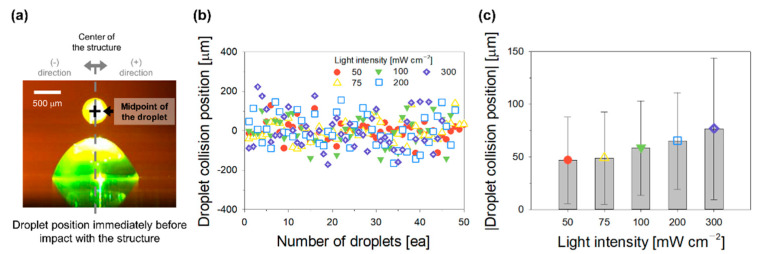
Investigation of droplet directionality. (**a**) Photograph acquired immediately before a droplet collided with the structure; (**b**) the distribution, (**c**) average, and standard deviation of droplet collision positions with respect to the light intensity.

The position of photocurable droplets immediately before the droplets impacted the structure was measured by examining images captured at 30,000 fps using a high-speed camera (Figure 2a). The (−) and (+) notations correspond to the impact occurring on the left and right sides of the structure, respectively. The positions (with respect to the center of the structure) of successively ejected 50 droplets under a specific light intensity were measured and shown in Figure 2b. The light intensity was set to 50, 75, 100, 200, and 300 mW cm^−2^. A light intensity of 50 mW cm^−2^ was the minimum to form the structure. Above 300 mW cm^−2^, the pneumatic printing system is prone to clogging due to high light energy. Fifty droplets can fabricate a main structure of a certain height after the formation of a seed structure. A linear structure could be readily constructed by adjusting only the Z-axis stage to maintain the standoff distance while the droplets were continuously ejected. The number of successively ejected droplets is placed on the X-axis of Figure 2b, and the position where each droplet collided with the structure is identified on the Y-axis using a separator (red circle, yellow triangle, green inverted triangle, blue square, and purple diamond, in increasing order of light intensity). For the light intensity of 50 mW cm^−2^, the position where the droplets collided with the structure was mostly within approximately ±50 μm from the center of the structure. As the light intensity increased from 50 to 300 mW cm^−2^, the number of droplets colliding at a position farther from the center of the structure increased. For the light intensity of 300 mW cm^−2^, the droplet position of the collision with the structure was distributed within approximately ±200 μm from the center of the structure. To analyze this more quantitatively, the distance between the midpoint of each droplet immediately before its collision with the structure and the center of the structure was measured (Figure 2c). As the light intensity increased (50, 75, 100, 200, and 300 mW cm^−2^), the average value (±standard deviation) of the distances increased (46.8 ± 41.0, 48.8 ± 43.8, 58.4 ± 44.9, 65.0 ± 45.8, and 76.6 ± 67.2 μm). Thus, the droplet directionality deteriorated with increasing light intensity. To clarify the effects of this phenomenon on the dimensions of the linear structure, the midpoints of the single layers formed after the droplet deposition on the structure was analyzed.

### 3.3. Shape and Position of Deposited Single Layers According to Droplet Directionality

**Figure 3:**
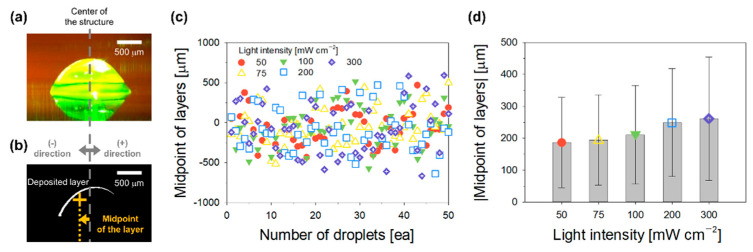
Analysis of the shape and position of deposited single layers. (**a**) Photograph of a deposited single layer acquired after the droplet–structure collision; (**b**) the morphology of the formed single layer; (**c**) the distribution; (**d**) average, and standard deviation of the midpoints of the deposited single layers as a function of light intensity.

The formation of each single layer immediately after a droplet collided with the structure was analyzed by focusing on the midpoints of the formed single layers. After a droplet collided with the structure, it spread along the surface of the structure, and the hydrogel precursor was polymerized by the transmitted guided light (Figure 3a). The upper part of the structure maintained a hemispherical shape. The morphology of the formed single layer was assessed by comparing the images of the structures before and after the droplet collision (Figure 3b). The (−) and (+) notations are based on the center of the structure and indicate the presence of the midpoints of the formed single layers on the left and right sides, respectively. The positions of approximately 50 droplets subjected to various light intensities were measured. The results indicated that the midpoints of the deposited single layers drifted further away from the center of the structure as the light intensity increased from 50 to 300 mW cm^−2^ (Figure 3c). To analyze this more quantitatively, the distance between the midpoint of the deposited single layers and the center of the structure was measured (Figure 3d). The results suggested that the mean value (±standard deviation) of the distances increased (186.6 ± 141.0, 194.1 ± 141.5, 210.8 ± 154.0, 249.1 ± 169.5, and 261.1 ± 193.0 μm) with increasing light intensity (50, 75, 100, 200, and 300 mW cm^−2^). In other words, as the light intensity increased, the droplet directionality deteriorated, which determined the position of the deposited single layers. Subsequently, the effects of the light-intensity-dependent changes in the position of the single layer on the formation of the final structure were investigated.

### 3.4. Formation of Final Structure by Successive Deposition of Single Layers

**Figure 4:**
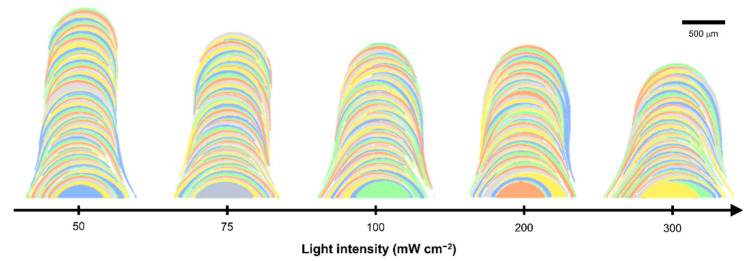
Final structures formed by successive deposition of single layers at different light intensities.

Each single layer formed by a droplet was marked with a different color to facilitate identification (Figure 4). As the light intensity increased, the dispersion of the droplet collision spots became larger; moreover, as described in Section 3.1, the average midpoints of the deposited single layers moved further away from the center of the structure. Consequently, the diameter of the structure increased, and the layer thickness per droplet decreased. Through these analyses, the effect of light intensity on the dimensions of structures is clearly identified, and ultimately, the dimensions of structures that can be fabricated and manipulated with the light-guided 3D printing method can be defined.

## 4. Conclusions

In this study, the light-intensity-dependent changes in the dimensions of a 3D-printed structure using a new in situ light-guided method were analyzed. As the light intensity increased from 50 to 300 mW cm^−2^, the average value (±standard deviation) of the distance between the midpoint of a droplet immediately before it collided with the structure and the center of the structure gradually increased from 46.8 ± 41.0 to 76.6 ± 67.2 μm, that is, the droplet directionality deteriorated. Additionally, the average value (±standard deviation) of the distance between the deposited single layer and the center of the structure gradually increased from 186.6 ± 141.0 to 261.1 ± 193.0 μm with increasing light intensity. Essentially, the instability of the ejected falling droplets increased and the droplet directionality deteriorated with increasing light intensity. This increased the dispersion of the droplet midpoints, causing the average midpoints of the deposited single layers to shift further away from the center of the structure. Consequently, the diameter of the main structure formed by successive layer-by-layer stacking increased, and the layer thickness per droplet decreased. 

## Data Availability

Not applicable.

## References

[1] Ibrahim D., Broilo T.L., Heitz C., de Oliveira M.G., de Oliveira H.W., Nobre S.M., Dos Santos Filho J.H., Silva D.N. (2009). Dimensional Error of Selective Laser Sintering, Three-Dimensional Printing and PolyJet Models in the Reproduction of Mandibular Anatomy. J. Craniomaxillofac. Surg..

[2] Castiaux A.D., Pinger C.W., Hayter E.A., Bunn M.E., Martin R.S., Spence D.M. (2019). PolyJet 3D-Printed Enclosed Microfluidic Channels without Photocurable Supports. Anal. Chem..

[3] Meess K.M., Izzo R.L., Dryjski M.L., Curl R.E., Harris L.M., Springer M., Siddiqui A.H., Rudin S., Ionita C.N. (2017). 3D Printed Abdominal Aortic Aneurysm Phantom for Image Guided Surgical Planning with a Patient Specific Fenestrated Endovascular Graft System. Proc. SPIE-Int. Soc. Opt. Eng..

[4] Liu T., Guessasma S., Zhu J., Zhang W., Nouri H., Belhabib S. (2018). Microstructural defects induced by stereolithography and related compressive behaviour of polymers. J. Mater. Process. Technol..

[5] Manapat J.Z., Chen Q., Ye P., Advincula R.C. (2017). 3D Printing of Polymer Nanocomposites via Stereolithography. Macromol. Mater. Eng..

[6] Melchels F.P., Feijen J., Grijpma D.W. (2010). A Review on Stereolithography and Its Applications in Biomedical Engineering. Biomaterials.

[7] Lee Y., Han J., Choi B., Yoon J., Park J., Kim Y., Lee J., Kim D.H., Kim D.M., Lim M. (2018). Three-Dimensionally Printed Micro-electromechanical Switches. ACS Appl. Mater. Interfaces.

[8] Leigh S.J., Bradley R.J., Purssell C.P., Billson D.R., Hutchins D.A. (2012). A Simple, Low-Cost Conductive Composite Material for 3D Printing of Electronic Sensors. PLoS ONE.

[9] Lim J., Kim J. (2020). 3D Vascular Replicas Composed of Elastomer–Hydrogel Skin Multilayers for Simulation of Endovascular Intervention. Adv. Funct. Mater..

[10] Lim J., Kim A.R., Kim S., Lee S., Yoo D., Park J., Kim J. (2019). A New Dip Coating Method Using Supporting Liquid for Forming Uniformly Thick Layers on Serpentine 3D Substrates. Adv. Mater. Interfaces.

[11] Lim J., Hwang J., Kim J. (2021). Rapid and Accurate Manufacture of 3D Vascular Replicas with Smooth Inner Surfaces Using Wax-Coated Molds. Adv. Mater. Technol..

[12] Msallem B., Sharma N., Cao S., Halbeisen F.S., Zeilhofer H.F., Thieringer F.M. (2020). Evaluation of the Dimensional Accuracy of 3D-Printed Anatomical Mandibular Models Using FFF, SLA, SLS, MJ, and BJ Printing Technology. J. Clin. Med..

[13] Tappa K., Jammalamadaka U. (2018). Novel Biomaterials Used in Medical 3D Printing Techniques. J. Funct. Biomater..

[14] Appleyard D. (2015). Powering up on Powder Technology. Met. Powder Rep..

[15] Karakurt I., Lin L. (2020). 3D printing technologies: Techniques, materials, and post-processing. Curr. Opin. Chem. Eng..

[16] Ligon S.C., Liska R., Stampfl J., Gurr M., Mulhaupt R. (2017). Polymers for 3D Printing and Customized Additive Manufacturing. Chem. Rev..

[17] Ionita C.N., Mokin M., Varble N., Bednarek D.R., Xiang J., Snyder K.V., Siddiqui A.H., Levy E.I., Meng H., Rudin S. (2014). Challenges and Limitations of Patient-Specific Vascular Phantom Fabrication Using 3D Polyjet Printing. Proc. SPIE Int. Soc. Opt. Eng..

[18] Hu K., Jin S., Wang C.C.L. (2015). Support slimming for single material based additive manufacturing. Comput. Aided Des..

[19] Wei C., Chueh Y.-H., Zhang X., Huang Y., Chen Q., Li L. (2019). Easy-To-Remove Composite Support Material and Procedure in Additive Manufacturing of Metallic Components Using Multiple Material Laser-Based Powder Bed Fusion. J. Manuf. Sci. Eng..

[20] Lim J., Kim Y.K., Won D.J., Choi I.H., Lee S., Kim J. (2019). 3D Printing of Freestanding Overhanging Structures Utilizing an In Situ Light Guide. Adv. Mater. Technol..

[21] Choi M., Choi J.W., Kim S., Nizamoglu S., Hahn S.K., Yun S.H. (2013). Light-guiding hydrogels for cell-based sensing and optogenetic synthesis in vivo. Nat. Photonics.

[22] Guo J., Liu X., Jiang N., Yetisen A.K., Yuk H., Yang C., Khademhosseini A., Zhao X., Yun S.H. (2016). Highly Stretchable, Strain Sensing Hydrogel Optical Fibers. Adv. Mater..

[23] Choi M., Humar M., Kim S., Yun S.H. (2015). Step-Index Optical Fiber Made of Biocompatible Hydrogels. Adv. Mater..

[24] Ciddor P.E. (1996). Refractive index of air: New equations for the visible and near infrared. Appl. Opt..

[25] Lim J., Lee S., Kim J. (2020). Structural dimensions depending on light intensity in a 3D printing method that utilizes in situ light as a guide. Micro Nano Syst. Lett..

[26] Choi I.H., Kim Y.K., Lee S., Lee S.H., Kim J. (2015). A Pneumatic Drop-On-Demand Printing System with an Extended Printable Liquid Range. J. Microelectromech. Syst..

[27] Agrawal M., Premlata A.R., Tripathi M.K., Karri B., Sahu K.C. (2017). Nonspherical Liquid Droplet Falling in Air. Phys. Rev. E..

[28] Balla M., Kumar Tripathi M., Sahu K.C. (2019). Shape oscillations of a nonspherical water droplet. Phys. Rev. E.

[29] Agrawal M., Katiyar R.K., Karri B., Sahu K.C. (2020). Experimental investigation of a nonspherical water droplet falling in air. Phys. Fluids.

[30] Zhang B., Ling Y., Tsai P.H., Wang A.B., Popinet S., Zaleski S. (2019). Short-term oscillation and falling dynamics for a water drop dripping in quiescent air. Phys. Rev. Fluids.

